# Specification of the patterning of a ductal tree during branching morphogenesis of the submandibular gland

**DOI:** 10.1038/s41598-020-79650-y

**Published:** 2021-01-11

**Authors:** Janice L. Walker, Weihao Wang, Edith Lin, Alison Romisher, Meghan P. Bouchie, Brigid Bleaken, A. Sue Menko, Maria A. Kukuruzinska

**Affiliations:** 1 Department of Pathology, Anatomy and Cell Biology, Thomas Jefferson University, 1020 Locust Street, Suite 564, Philadelphia, PA 19107 USA; 2 Department of Translational Dental Medicine, School of Dental Medicine, Boston University, 700 Albany Street, W201, Boston, MA 02118 USA

**Keywords:** Developmental biology, Morphogenesis

## Abstract

The development of ductal structures during branching morphogenesis relies on signals that specify ductal progenitors to set up a pattern for the ductal network. Here, we identify cellular asymmetries defined by the F-actin cytoskeleton and the cell adhesion protein ZO-1 as the earliest determinants of duct specification in the embryonic submandibular gland (SMG). Apical polarity protein aPKCζ is then recruited to the sites of asymmetry in a ZO-1-dependent manner and collaborates with ROCK signaling to set up apical-basal polarity of ductal progenitors and further define the path of duct specification. Moreover, the motor protein myosin IIB, a mediator of mechanical force transmission along actin filaments, becomes localized to vertices linking the apical domains of multiple ductal epithelial cells during the formation of ductal lumens and drives duct maturation. These studies identify cytoskeletal, junctional and polarity proteins as the early determinants of duct specification and the patterning of a ductal tree during branching morphogenesis of the SMG.

## Introduction

Epithelial tissues with exocrine function, including the mammary gland, the lung, and the salivary gland, attain their mature structure and function through the complex developmental process of branching morphogenesis (reviewed in^1^). The salivary submandibular gland (SMG) utilizes the process of clefting to drive branching morphogenesis that culminates in the establishment of an array of ducts terminating in secretory acini. As such, the SMG has served as a template for deciphering the cellular and molecular events underlying the development of a gland from a single epithelial bud into a highly branched structure^1,2^. The complexity of the SMG morphogenetic process is underscored by its dependence on cellular rearrangements, patterned actomyosin contractility, dynamic interactions with different cell types and their extracellular matrix (ECM) environments, and by the induction of gene expression programs that regulate region-specific cell dynamics^3–13^. Furthermore, recent global transcriptomic and epigenetic mapping of salivary gland development and regulation have revealed additional insights into the mechanisms underlying its development and function^14,15^.

During the past decade, numerous studies have focused on the identification of salivary progenitor cells, their molecular features and regulation^5,9,16–18^. Early in branching morphogenesis, the SMG comprises proximal and distal progenitors defined by their location and expression of immature cell markers. One population of proximal progenitor cells, localized in the vicinity of the epithelial stalk region, expresses keratin 5 (K5) intermediate filaments and Sox2^16,18^. These proximal progenitors have been shown to generate higher order ductal structures^19^ and their patterning requires the expression of the Hippo pathway effector, YAP^3^. The maintenance of ductal progenitors requires vasoactive intestinal peptide (VIP) signals from the adjacent neuronal system^5^ and epiregulin^3^ and involves the activities of a cyclic AMP/protein kinase A pathway^7^. In contrast, distal progenitors at the end of the expanding bud regions express keratin 14 (K14), Kit, FGFR2b and Sox10^17^, and they mark proacinar cells^19,20^. The establishment of these distal progenitors has been shown to require the expression of Sox9 and to depend on Fgf10 signaling from the adjacent mesenchyme^21^, with KIT^+^ progenitor expansion depending on exosomal transport of miR-133b-3p from mesenchyme^22^. As cytodifferentiation progresses, the end bud progenitors also express Myb, shown to be regulated by Wnt signaling that suppresses Kit and promotes acinar cell differentiation^19^. Acinar differentiation is also marked by the expression of Mist1^23^. Despite these insights, there is a considerable gap in the understanding of the mechanisms by which ductal progenitor cells acquire polarity, participate in specification of the path of ductal extension and reorganize to form higher order branched and differentiated salivary gland ductal structures.

Our earlier studies with mouse SMGs showed that the patterns of acinar and ductal cell fates are already established at the initial bud stage at embryonic day E12.5, and that they are maintained throughout branching morphogenesis^20^. We found that acinar progenitors are restricted to the peripheral cell layer in contact with the basement membrane and require the cell–cell adhesion receptor E-cadherin for the establishment of apical-basal polarity and for coordinating acinar cell proliferation with new bud formation. In contrast, the proliferating polymorphous cells in the interior regions of terminal buds are comprised of ductal progenitors. Nonetheless, how a subpopulation of polymorphous cells in the developing SMG bud establishes asymmetry, reorganizes the ductal progenitor cells along the proximal–distal axis and extends this process into the newly forming buds, thus establishing the pattern for the ductal tree, remains unknown.

Our previous studies have shown that proximal regions of the terminal buds are sites of presumptive duct formation^20^. As the initial bud undergoes clefting to form new buds at E13.5, cells in their proximal regions are subject to dramatic morphogenetic changes to form early ductal structures. These cells are characterized by prominent staining of cortical filamentous actin (F-actin), and they mark extensions of the newly forming ducts leading to the inner bud regions prior to apical/basal polarization of the ductal progenitor cells and the subsequent appearance of ductal lumens. As the ducts extend they assemble mature E-cadherin junctions, the duct-forming cells withdraw from the cell cycle, and E-cadherin is essential to survival of these cells during duct maturation^20^.

Recent studies have contributed significant insights into signals that drive duct development and maturation. They have shown that branching morphogenesis involves specialization of epithelial progenitors into distal and proximal, with proximal progenitors giving rise to higher order ducts^21^. Moreover, ductal tubulogenesis has been shown to require neuronal-epithelial communication mediated, in part, by Wnt signals from epithelial progenitors. The latter are required for neuronal cell survival, proliferation and for the maintenance of progenitor cells^9^, where neuronal enervation during branching morphogenesis contributes signals that guide ductal tubulogenesis of the SMG^5^. These studies reveal the critical interaction between neuronal ganglion-secreted VIP and the developing SMG epithelium to drive duct formation by promoting the fusion of microlumens, central to the process of formation of a contiguous ductal lumen^7^. Nonetheless, the earlier events required for specification of the patterning of the ductal tree and the formation of nascent ductal structures have not been elucidated.

Many fundamental insights into the molecular and cellular events underlying SMG embryonic development have been generated using embryonic SMG explant cultures ex vivo that mimic developmental events in vivo^2,5,7–9,16,20,24–26^. In this study, we have used this ex vivo SMG explant culture system to identify the initial mechanisms driving formation of ductal structures at the earliest stages of SMG branching morphogenesis and to reveal the cellular and molecular basis of ductal cell specification at the single bud stage before any ductal markers are detected. This approach allowed us to follow cellular reorganizations into ducts along spatial and temporal coordinates to identify the molecular determinants of duct extension into the developing buds and to elucidate the mechanisms underlying the salivary gland branching program. We now show that reorganization of ductal progenitors into ductal structures in the developing embryonic SMG involves the establishment of single-cell asymmetries (SCAs) that become expanded to mark prospective ductal regions. We identify F-actin and ZO-1 as the early determinants of cellular asymmetry and demonstrate functional roles for the cellular processes that organize immature duct cells into tubes along an axis that extends the developing duct into the newly forming buds. Our studies demonstrate that ZO-1 recruits a polarity protein, PKCζ, to the apical regions of ductal progenitor cells, which functions together with basal polarity signals involving ROCK signaling to set up apical/basal polarity of the ductal epithelial cells that now pattern the path of duct extension in the inner bud region. These cells are then organized into mature ducts in a process requiring myosin II-dependent mechanical forces. Collectively, these findings generate the first temporal map of cytoskeletal, polarity, signaling and mechanosensing proteins involved in duct elongation to produce a ductal tree during branching morphogenesis of the SMG.

## Results

### ZO-1 sets up asymmetry across the field of ductal progenitors

**Figure 1:**
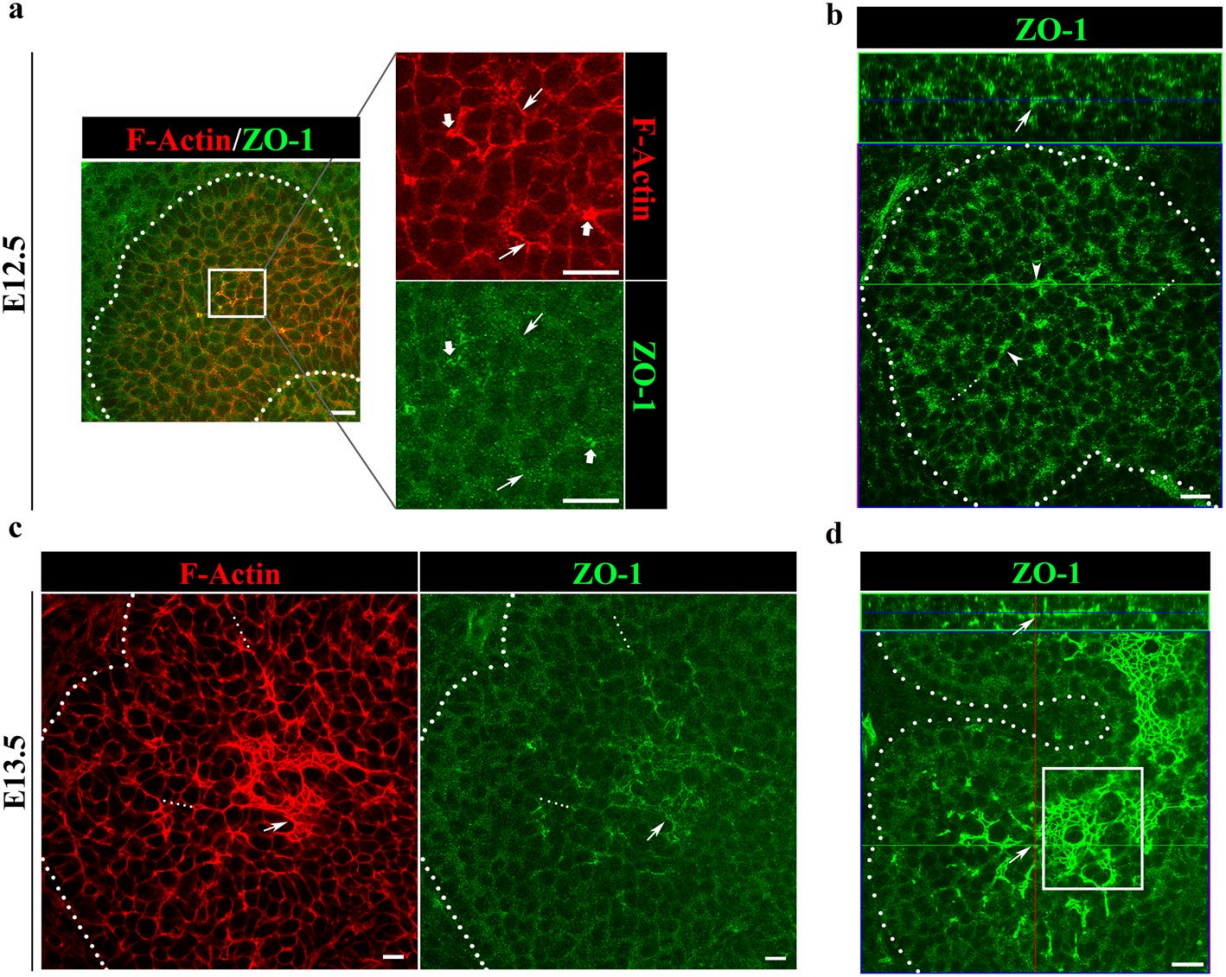
F-actin and ZO-1 set up a roadmap for prospective apical domains in cells destined for ductal lineage. (**a**) Immunofluorescence of paraformaldehyde-fixed SMG explants shows preferential co-localization of F-actin and ZO-1 to one membrane domain in the ductal progenitor cells at the single bud stage, with F-actin delineating converging SCAs (boxed area, → *and* ➔). Merged image size bar represents 10 µm, while split images scale bars are 20 µm. (**b**) Acetone/methanol-fixed tissues prior to immunofluorescence staining revealed that a single bud with three clefts displayed asymmetric distribution of ZO-1 which marked future paths for duct extension. (**c**) Immunofluorescence of paraformaldehyde-fixed E13.5 SMG explants showed that distal regions of buds, defined by the newly formed clefts, displayed ductal paths enriched for F-actin and ZO-1 (*…*). Arrows (→) indicate F-actin-enhanced cellular domains with paucity of ZO-1, suggesting that F-actin precedes ZO-1. (**d**) Acetone/methanol-fixed E13.5 SMG buds display proximal regions (boxed area, →) with strong ZO-1 staining across the expanding field of asymmetry-enriched cells. Size bars, 20 µm. Results are representative of 6 independent experiments (n = 8/group).

Previously, we showed that cells in the proximal bud regions at early stages of SMG development undergo dramatic rearrangements to form ductal structures that are demarcated by F-actin^20^. We now examined F-actin localization at the single bud stage, E12.5, and found that F-actin was preferentially associated with a single membrane domain of individual cells within the bud, which we refer to as defining “single cell asymmetry” (SCA) (Fig. 1a, boxed area, left panel; and arrows, top right panel). In some regions, F-actin was concentrated at points of converging SCAs (Fig. 1a, arrowheads), similar to vertices formed during axis elongation and cellular rearrangements in Drosophila^27^. By E13.5, F-actin staining expanded across a field of cells in more proximal regions of the developing buds (Fig. 1c, E13.5, arrow) while also delineating the prominent extensions of established SCAs that marked developing ductal paths into the newly forming buds defined by initiated clefts (Fig. 1c, dotted lines, left panel). These results suggest that duct elongation occurred via cellular reorganization in the inner bud regions in a manner resembling larval axis elongation via the planar cell polarity pathway in *Drosophila*, with a prominent role for the actin cytoskeleton^28^.

Classical axis elongation also involves the recruitment of polarity proteins to sites marked by F-actin to establish cellular asymmetry^27^. We searched for a polarity protein expressed prior to the formation of ductal structures that could link to F-actin at the sites of single cell asymmetries in the proximal regions of inner buds by interrogating the salivary gland atlas (http://sgmap.nidcr.nih.gov/sgmap/sgexp.html). The atlas showed that message for the tight junction protein ZO-1 was expressed from E11.5, the earliest stage of SMG development. This finding, together with the ability of ZO-1 to bind directly to F-actin, made this polarity protein a strong candidate for a molecular regulator of duct specification^29,30^. Immunolocalization studies at E12.5, the initial bud stage, coupled with high-resolution confocal imaging, confirmed that ZO-1 was present in a subset of polymorphous ductal progenitors in the middle of the bud along the cellular domains where F-actin established SCAs (Fig. 1b, arrows). Different from F-actin, ZO-1 labeling along these asymmetries was often punctate, broad in distribution or discontinuous (Fig. 1b,c, arrows and arrowheads). The presence of F-actin SCAs on cells in the distal duct-forming region of the SMG bud extended beyond the region were ZO-1 could be detected (Fig. 1c, dotted lines) suggesting that the asymmetric organization of F-actin preceded the recruitment of ZO-1 to these sites. However, the pattern of ZO-1 localization suggested that, together with F-actin, ZO-1 marked the paths for prospective duct elongation from the stalk region. This conclusion was based on studies with E12.5 SMGs where the initial bud had already begun to form clefts (Fig. 1b, arrows, dotted lines). Here, ZO-1 exhibited a multipronged pattern pointing in the direction of the prospective ductal paths of what would become three newly forming buds. These results suggest that F-actin sets up single-cell asymmetries to specify the apical domains of presumptive duct-forming cells and, together with ZO-1, identifies the direction of duct extension as early as the initial bud stage.

Co-labeling for ZO-1 and F-actin in E13.5 SMGs demonstrated the prominent asymmetric distribution of ZO-1 within the proximal bud region and the alignment of this polarity protein along an axis delineated by F-actin (Fig. 1c). Together, F-actin and ZO-1 appeared to define the path for extending and expanding the ductal tree. At E13.5, cells more distal to the cells marked by both F-actin and ZO-1, displayed asymmetrically localized F-actin without ZO-1 (Fig. 1c), providing supportive evidence that F-actin recruited ZO-1 to SCAs. It is important to note that at E13.5 of SMG morphogenesis the asymmetric distribution of ZO-1 along apical domains of duct-forming cells had expanded across the field of ductal progenitors in the proximal regions of buds with well-defined and advanced clefts (Fig. 1d, E13.5, boxed area). When viewed in the orthogonal, the prospective ductal network defined by F-actin and ZO-1 formed along one tissue domain within the center of the SMG bud, showing that these cells were organized along a proximal–distal axis within the bud (Fig. 1d, orthogonal, arrow). Collectively, these findings suggest that ZO-1 collaborates with F-actin in setting up cellular asymmetry as an initial step in the process of duct specification.

**Figure 2:**
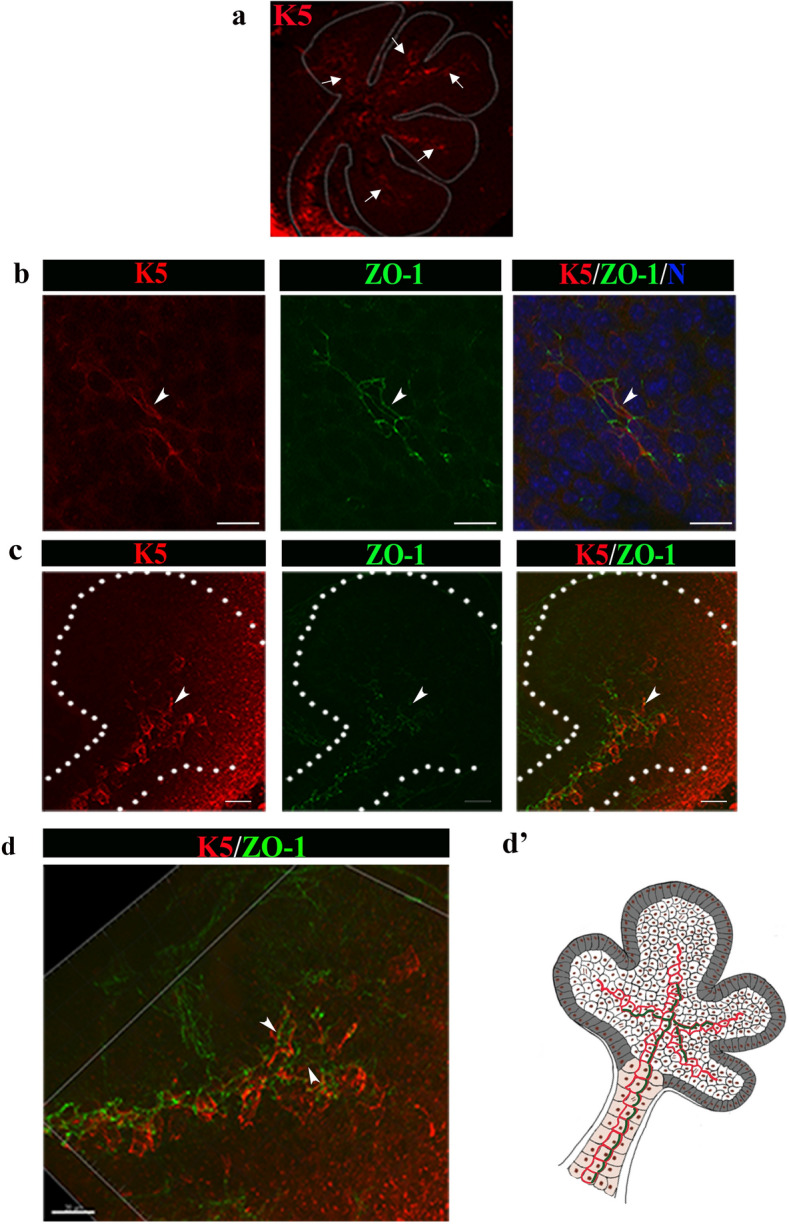
F-actin and ZO-1 establish asymmetry in K5 progenitor cells. (**a**) In addition to marking the ductal path in proximal bud region, extending ducts in E13.5 SMGs comprised K5 + cells at distal sites (→). (**b**) K5 + cells (red, →) in the proximal bud exhibit ZO-1 asymmetries (green, →); nuclei are marked by the blue TOPRO-3 stain. (**c**) K5+ cells in the proximal region of the sublingual gland displayed ZO-1 at sites of asymmetry (→). (**d**) 3D volumetric rendering with Imaris software of the sublingual gland in (**c**) revealed the organization of K5 + ductal progenitors relative to their ZO-1 asymmetries in the proximal region of the forming ductal tree (→)*.* Size bars, 20 µm. (**d’**) Schematic representation of K5 + cells with ZO-1 asymmetries in the developing ductal regions of the salivary gland in (**a**–**d**, red, green). Results show representative images of 5 experiments (n = 10/experiment).

Expression of the cytokeratin K5 is a defining feature of epithelial progenitor cells in a number of tissues including the SMG duct^5,16^. In particular, K5+ epithelial progenitors produce Wnt, a signal that promotes gangliogenesis in the SMG^9^, where the latter has an important inductive role in duct formation^7^. Thus, we examined whether K5+ ductal progenitor cells acquired SCAs to set up the duct-forming region at early stages of SMG development. We found that K5+ cells localized along the ductal tree by E13.5 (Fig. 2a, arrows). Co-labeling for K5 and ZO-1 showed that ZO-1 asymmetries localized to K5+ cells in the duct forming region of the proximal bud (Fig. 2b, arrows). We also found that K5 marked single cell asymmetries in the sublingual gland (Fig. 2c). Volumetric rendering of the image in Fig. 2c in three dimensions showed that K5+ ductal progenitors were asymmetrically labeled with ZO-1 at their apical domains (Fig. 2d, schematic Fig. 2d’). This suggested that F-actin/ZO-1 junctions drove the establishment of cellular asymmetries in the subpopulation of K5 + ductal progenitors, which were then expanded across a field of cells to coordinate ductal branching with clefting for new bud formation (schematic, Fig. 2d’). Furthermore, since F-actin preceded ZO-1 at the sites of asymmetry, it likely functioned upstream of ZO-1 in organizing cell polarity. Taken together, these data suggested a novel function for ZO-1 in specifying ductal cells by collaborating with F-actin to set up SCAs and to coordinate organization and orientation of ductal progenitor cells within the plane of the SMG tissue for duct formation.

### ZO-1 is required for ductal extension into the bud

**Figure 3:**
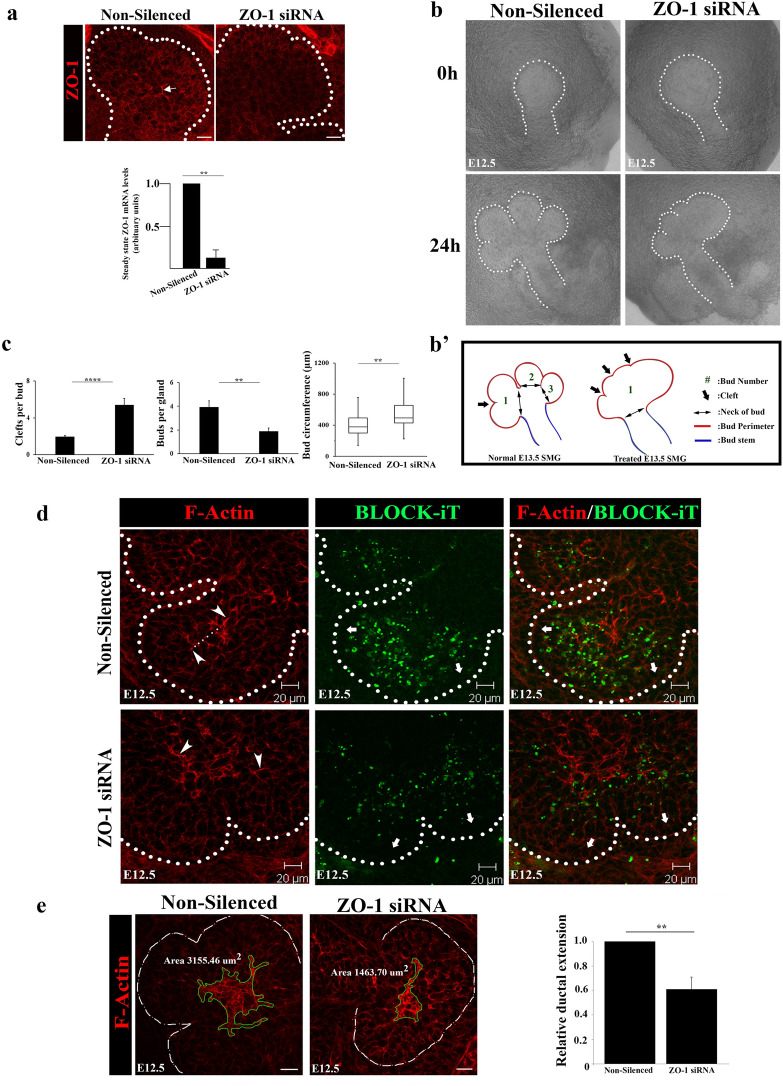
ZO-1 maintains F-actin at the sites of asymmetry and is required for duct extension early in SMG development. (**a**) Immunofluorescence staining of ZO-1 (→) in E12.5 SMG following ZO-1 knockdown with siRNA revealed loss of ZO-1-specific staining (n = 16). Bar graph of siRNA-mediated inhibition of ZO-1 in E12.5 SMG showed a significant, more than 80%, knockdown of the ZO-1 transcript levels (*p* ≤ 0.01). (**b**) Bright field images in Non-silenced (scrambled sequences) and ZO-1 siRNA-treated SMGs show inhibition of cleft progression and gland expansion. (**b’**) A schematic of observed changes in bud morphologies used for quantification of ZO-1-inhibited glands in (**c**), with marked (→) clefts and maturing buds. (**c**) Quantification of defects in branching morphogenesis following ZO-1 knockdown in E12.5 SMGs. Bar graphs and a box plot of non-silenced and ZO-1 siRNA-treated E12.5 SMGs revealed significant changes in gland morphologies, including increased number of clefts (*p* ≤ 0.0001) along with reduced bud numbers (*p* ≤ 0.01) and elevated bud circumference (*p* ≤ 0.01) in ZO-1-inhibited glands. (**d**) Immunofluorescence images depict inappropriately bundled F-actin coincident with the loss of ductal extensions in the absence of ZO-1 (→). BLOCK-iT marks siRNA uptake by the SMG with paucity of siRNA in cells in the outer layers of buds with columnar morphologies (➔). (**e**) Quantification of the effects of ZO-1 siRNA on F-actin expansion (areas defined by green outlines) during ductal extension. Bar graph indicates significant (*p* ≤ 0.01) reduction in the total area of F-actin upon ZO-1 inhibition. Size bars, 20 µm.

To confirm the early role of ZO-1 in setting up cellular asymmetries for the formation of new ductal structures, we inhibited ZO-1 expression with siRNA in E12.5 SMGs and examined the consequences on duct extension after 24 h in culture. Quantitative RT-PCR revealed a 90% knockdown of ZO-1 mRNA levels in siRNA-treated glands compared to controls (Fig. 3a, bar graph). This was confirmed by a statistically significant loss of ZO-1 labeling by immunofluorescence (Fig. 3a, arrow). SMGs treated with ZO-1 siRNA at E12.5 exhibited morphological aberrations within 24 h involving a flattening of the proximal duct-forming region with shallow initial clefts (Fig. 3b and schematic Fig. 3b’), indicating perturbation of the branching process in the absence of ZO-1. Quantification of changes in morphologies of ZO-1-inhibited glands confirmed an increased number of clefts per bud that coincided with a greater bud circumference and substantial reduction in bud numbers (n = 16/group) (Fig. 3c & schematic Fig. 3b’). To identify cells targeted by the ZO-1 siRNA and to determine the transfection efficiency, we co-transfected SMGs with BLOCK-iT™, a FITC-conjugated oligo reagent. Transfection-competent cells take up the siRNA (or scrambled control, Non-Silenced) and the BLOCK-iT reagent. The results confirmed that BLOCK-iT was effectively taken up by the inner bud cells including the subpopulation of ductal progenitors (Fig. 3d). Interestingly, there was a much lower level of uptake in the columnar cells of the outer cell layer in contact with the basement membrane, a population shown previously by us to be acinar progenitors^20^, and in mature ducts (Fig. 3d, block arrows). This preferential uptake allowed us to perturb expression of ZO-1 in immature duct cells without significantly affecting either acinar progenitors or differentiated duct cells. To determine how silencing of ZO-1 impacted F-actin organization along the ductal paths, we quantified areas of F-actin distribution and found that in the absence of ZO-1 F-actin organization was significantly constrained to ~ 50% of the proximal bud area with concomitant loss of extensions (Fig. 3d, arrowheads and dotted lines, and 3e, bar graph). Partial loss of ZO-1 resulted in a failure of F-actin to delineate and extend ducts into the more proximal regions of the inner bud (Fig. 3d). SMGs co-transfected with BLOCK-iT and non-silencing control siRNA had normal ductal structures delineated by F-actin that extended into the buds even in regions where BLOCK-iT was prominent (Fig. 3d, Non-Silenced, dotted lines).

**Figure 4:**
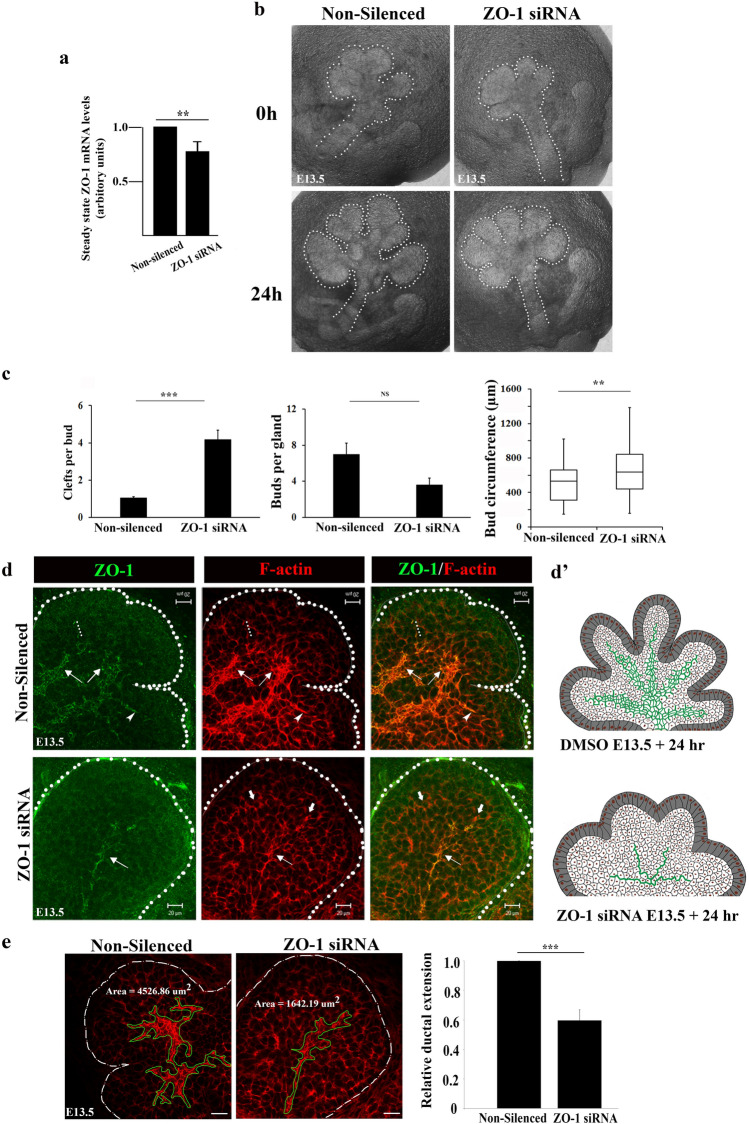
ZO-1 is required for F-actin expansion and extension into new buds. (**a**) Bar graph of siRNA-mediated inhibition of ZO-1 in E13.5 SMGs revealed a significant 20% knockdown of the transcript level (*p* ≤ 0.01). (**b**) Bright field images of non-silenced and ZO-1 siRNA-treated E13.5 SMGs showed inhibition of cleft progression and gland expansion. (**c**) Quantification of changes in E13.5 SMG branching morphogenesis (n = 12) following ZO-1 knockdown revealed significant increases in the number of clefts (*p* ≤ 0.001) and reduced bud numbers (*p* ≤ 0.01) concomitant with elevated bud circumference (*p* ≤ 0.01). (**d**) Immunofluorescence staining of ZO-1 (green) demonstrates the requirement for the expansion of F-actin (red) and asymmetric distribution in ductal progenitors (→ ,…). (**d’**) A schematic depicting the effects of ZO-1 inhibition on ductal tree patterning and branching morphogenesis. Arrows ( →) indicate loss of ductal extension in distal bud regions after siRNA-mediated ZO-1 depletion. Size bars, 20 µm. (**e**) Quantification of the area marking F-actin distribution (green outlines) in response to ZO-1 siRNA revealed significant inhibition (*p* ≤ 0.001) of F-actin-mediated ductal expansion and extension into new buds (bar graph).

We next examined the effect of inhibiting ZO-1 expression with siRNA at E13.5, when F-actin and ZO-1 already delineate early ductal structures that extend and expand across the proximal bud field. At this more developmentally advanced stage, treatment with siRNA specific to ZO-1 resulted in a 20% inhibition in the ZO-1 mRNA steady-state levels (Fig. 4a). Nonetheless, even 20% inhibition of ZO-1 caused defects in branching morphogenesis, characterized by buds with increased number of clefts that failed to progress to form mature buds (Fig. 4b). This coincided with augmented circumference and diminished number of maturing buds per gland (Fig. 4b,c). Further, immunostaining analyses revealed loss of both F-actin and ZO-1 expansions in more distal regions of the bud in the absence of normal clefting and budding (Fig. 4d, arrows and schematic Fig. 4d’). Indeed, co-labeling for ZO-1 and F-actin demonstrated an inhibition of directed duct extension into more distal regions of the developing buds in the absence of ZO-1 compared to non-silenced controls (Fig. 4d, Non-Silenced, dotted lines, arrowhead). Significantly, these distal regions of the buds devoid of ZO-1 in siRNA-treated glands displayed disorganized accumulation of F-actin although still with SCAs (Fig. 4d, ZO1-siRNA, block arrows). Quantification of F-actin distribution along the ductal paths in non-silenced and silenced glands revealed a 40% lesser expansion in the absence of ZO-1 (Fig. 4e, bar graph). Thus, loss of ZO-1 caused defects in ductal expansion and extension.

### aPKCζ is recruited to apical domains specified by ZO-1/F-actin and required for duct maturation

**Figure 5:**
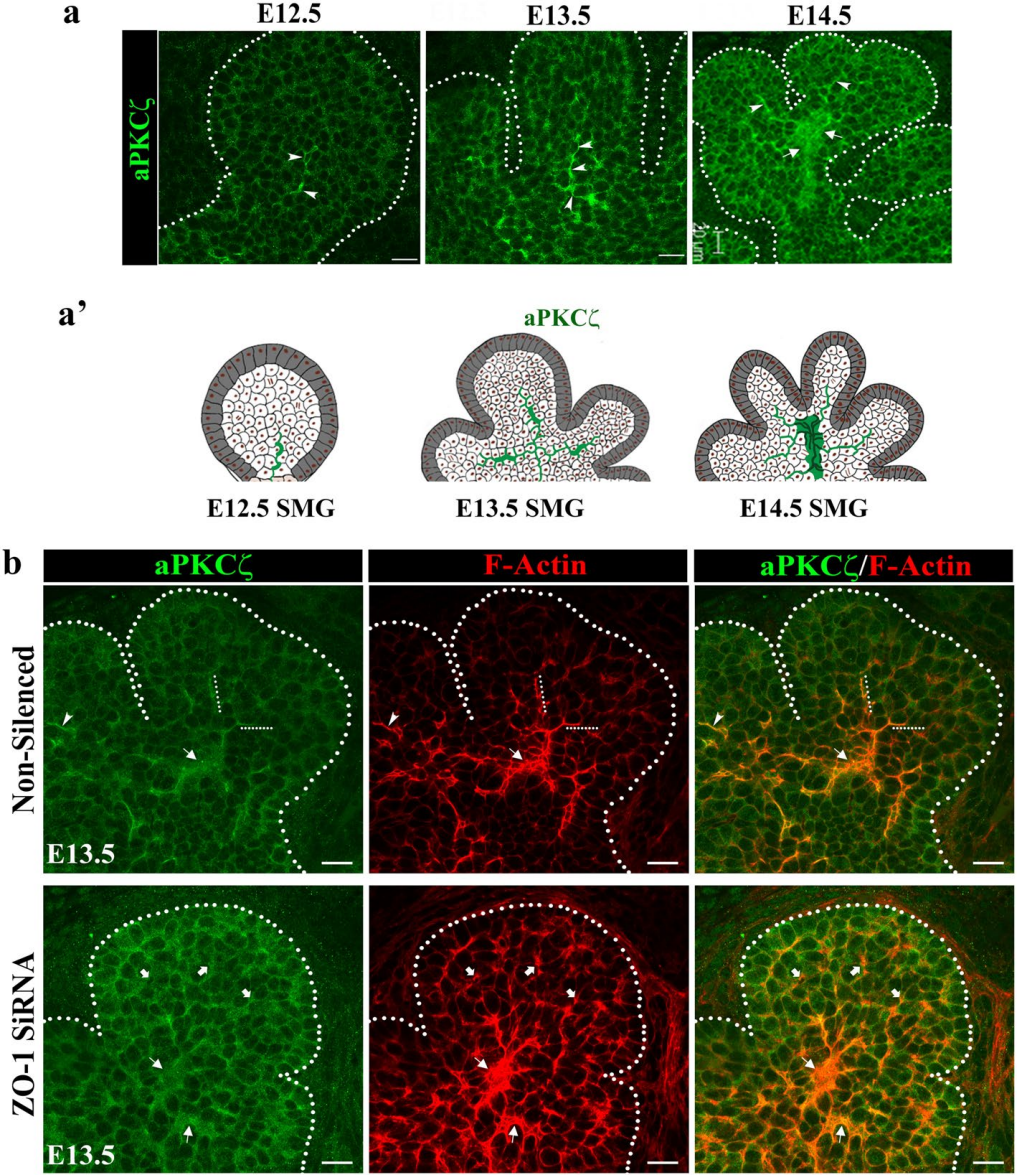
A polarity protein aPKCζ is recruited to the sites of asymmetry in the developing ducts in a ZO-1-dependent manner. (**a**) Immunofluorescence imaging of aPKCζ at progressive stages of branching morphogenesis from the single bud stage at E12.5 to a more elaborate multi-budded structure at E14.5. Arrows (→) point to initial enriched localization at single membrane domains marking future ductal extensions at E12.5, followed by a prominent distribution of aPKCζ to the sites of cellular asymmetry along the ductal paths that extend into the newly formed buds at E13.5. There was a further enhancement of aPKCζ in the developing duct regions that had acquired multi-cellular organization in E14.5 SMGs, as well as at the single membrane domains marking extending ductal paths into the newly forming buds (→). (**a’**) Schematic series representing contributions of aPKCζ to the developing ductal paths at different stages of branching morphogenesis. (**b**) Inhibition of ZO-1 expression with siRNA at E13.5 for 24 h resulted in the loss of F-actin-demarcated ductal extensions compared to the non-silenced controls (→). Instead, F-actin was bundled in the proximal regions of the newly formed buds with and apparent loss of cells enriched in aPKCζ at the sites of asymmetry ( →). Size bars, 20 µm. Results are representative of 5 independent experiments (n = 6/group).

So far, our studies have shown that the establishment of SCAs in ductal progenitor cells by F-actin and ZO-1 specifies the path for duct extension. In order to form ductal structures these progenitor cells must rearrange and acquire a polarized phenotype^28,31,32^. As this is typically achieved through the function of polarity proteins, we examined the spatial–temporal distribution of the polarity complex member aPKCζ from early stages of duct cell specification at the single bud stage E12.5, through to duct formation at E14.5^33,34^. To determine whether aPKCζ was recruited to duct forming regions and its temporal sequence relative to the time of duct specification by F-actin and ZO-1, we examined the localization and distribution of aPKCζ by immunofluorescence imaging. At E12.5, when ZO-1 and F-actin had already defined SCAs within the single bud and the path for duct extension (Fig. 1a), aPKCζ was detected along single cell domains in a small subpopulation of cells within the bud (Fig. 5a, E12.5, arrowheads and schematic Fig. 5a’). By E13.5 of SMG development, asymmetrically localized PKCζ had begun to expand across the field of cells in the duct forming regions of the bud (Fig. 5a, E13.5, arrowheads & schematic Fig. 5a’), although with a more limited distribution than F-actin or ZO-1 (see Fig. 4d Non-Silenced). At a later stage of development, E14.5, aPKCζ prominently marked expanded ductal structures with SCAs extending into newly forming buds (Fig. 5a, E14.5 block arrows and arrowheads, and schematic Fig. 5a’) and highly coincident with that of F-actin (Fig. 5b).

We next investigated whether ZO-1 was required for the localization of aPKCζ to the duct forming region of the SMG bud within the E13.5–E14.5 developmental window by inhibiting ZO-1 expression using siRNA. Results showed that siRNA knockdown of ZO-1 in E13.5 SMGs followed by culture for 24 h blocked the directed recruitment of aPKCζ to F-actin-rich regions in the developing bud (Fig. 5b, ZO-1 siRNA, arrows and block arrows). Inhibition of ZO-1, however, did not impact expression of aPKCζ, suggesting that the presence of ZO-1 at the sites of cellular asymmetries was a prerequisite for the recruitment of aPKCζ (Fig. 5b). These data suggest an early role for F-actin and ZO-1 in establishing asymmetries in future duct cells, followed by recruitment of aPKCζ, to ductal progenitor cells as they coalesce and extend into the bud region to organize into ductal structures.

### Myosin II drives ductal cell rearrangements into ductal structures

**Figure 6:**
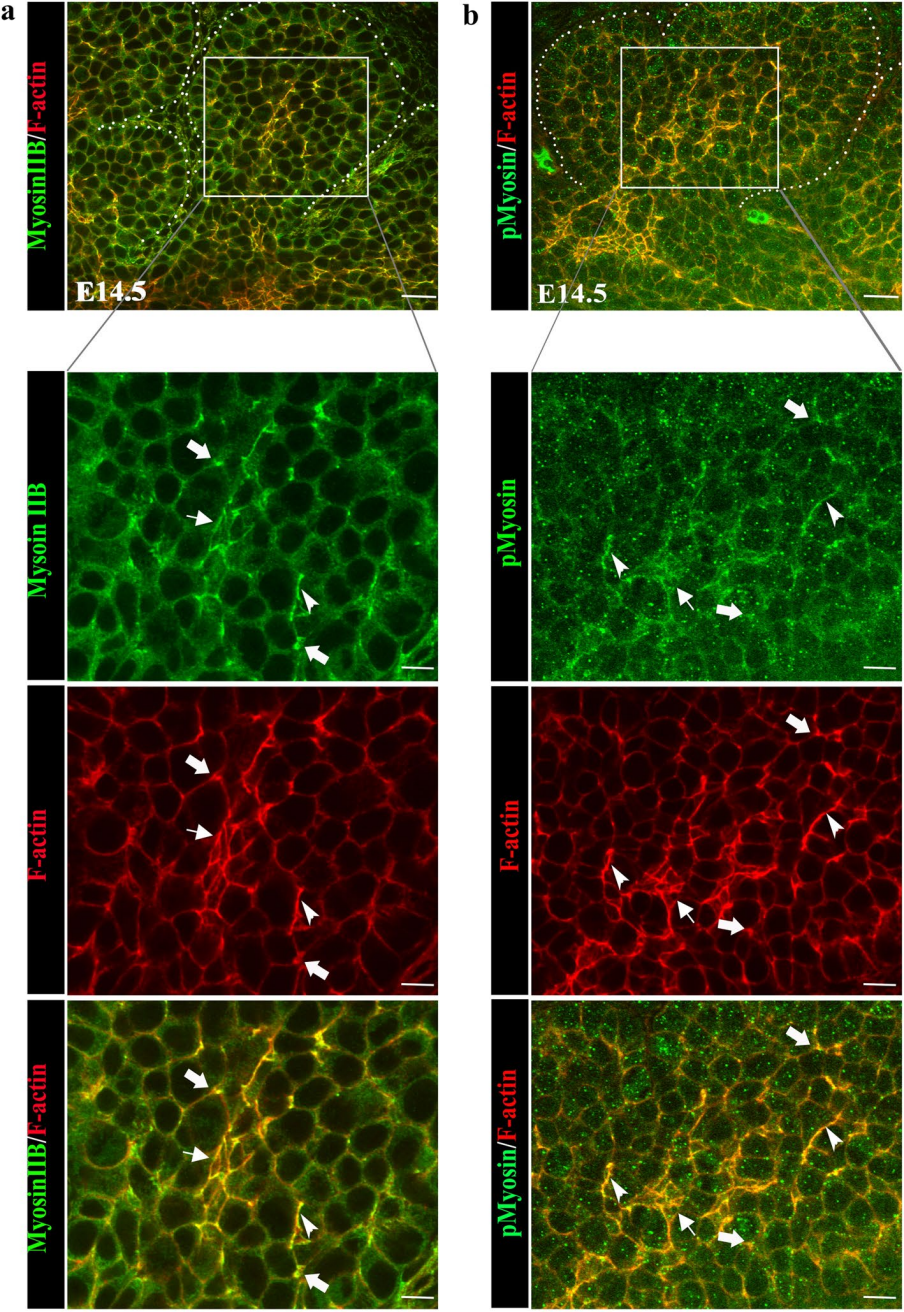
Myosin IIB participates in the mapping of the ductal tree. (**a**) Immunofluorescence staining for myosin IIB at E14.5 revealed a robust co-localization with F-actin in cells organizing into ductal structures (boxed area). Details of the region marked by a boxed area is shown in panels below. Myosin IIB (green) displayed a prominent punctate distribution at the apical domains of reorganizing ductal cells ( →), with F-actin (red) demarcating newly forming ductal paths. (**b**) Active myosin IIB, phospho-myosin IIB, was co-localized with F-actin to the sites of asymmetry in the extending proximal ductal regions (boxed area). Region marked by the square is shown in detail in a panel below. Phospho-myosin IIB (green) was broadly distributed at the apical domains of reorganizing ductal cells along the F-actin-marked ductal paths ( →). Size bars, 20 μm. Images are representative of 5 independent experiments (n = 8/group).

Extension of three-dimensional epithelial structures has been shown to involve the function of the motor protein myosin II, which controls planar cell intercalation, axis elongation, and establishment of tissue cytoarchitecture^28,35,36^. Myosin II produces tension along actin filaments, an important factor for both the assembly and maintenance of the ductal network. The high duty ratio of the myosin II isoform allows this myosin motor to maintain tension on actin filaments. Immunolabeling of E14.5 SMGs for myosin IIB revealed increased alignment along the actin filament network with prominent localization along cell borders leading to vertex-like structures (Fig. 6a, arrows and arrowheads)^37,38^. This finding is consistent with a mechanical role for myosin II in apical domain constriction and tension maintenance, providing the force necessary to execute the cellular arrangements required for formation of ductal lumens. Immunolabeling with antibody to the activated form of myosin II, p-myosin, at E14.5 verified that active myosin II functioned along actin filaments during the process of duct formation (Fig. 6b, arrows and arrowheads).

### Rho kinase (ROCK) regulates temporal events in duct formation but not duct specification

**Figure 7:**
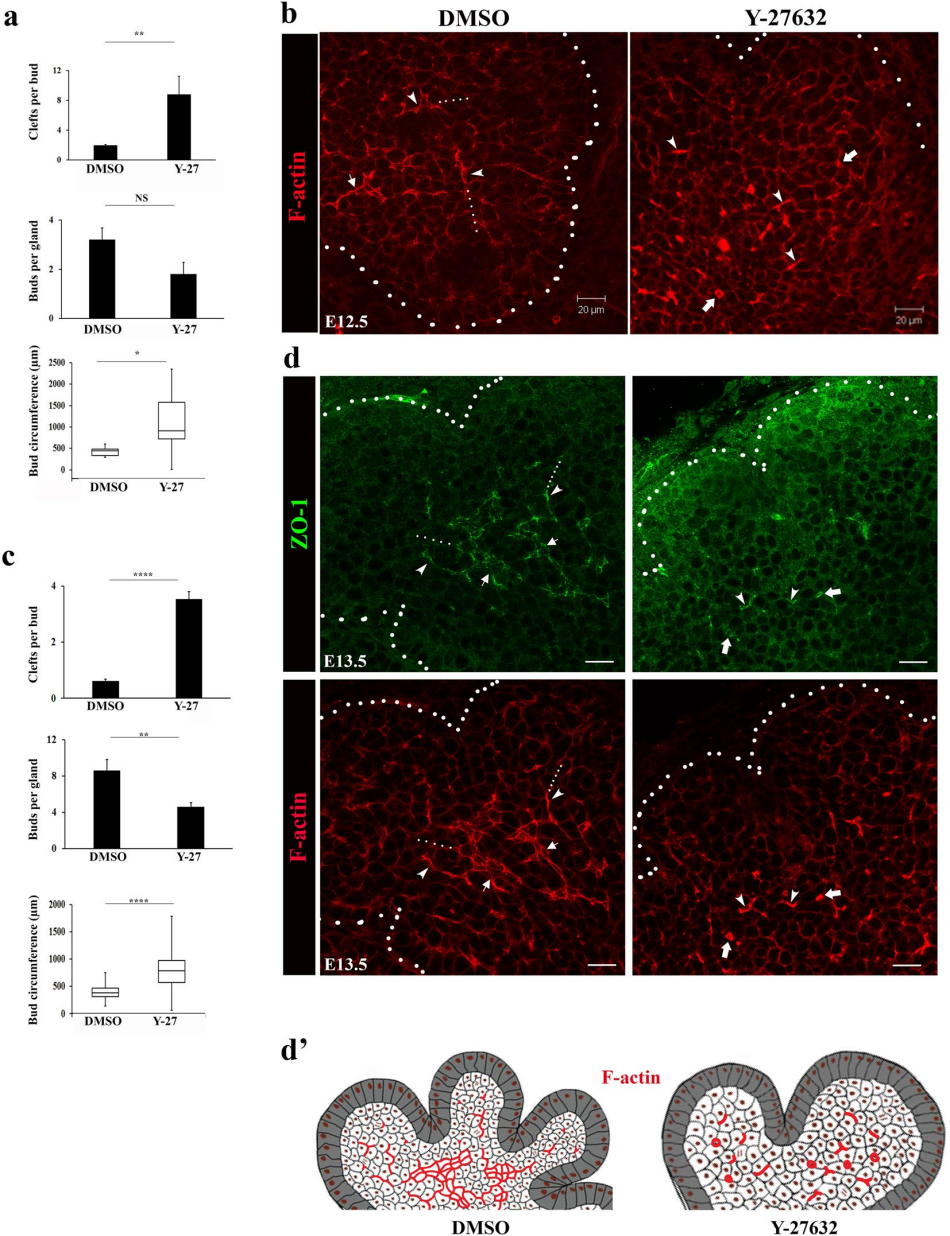
Inhibition of Rho kinase with Y-27632 disrupts the formation of F-actin marked ductal extensions. (**a**) Quantification of the effects of Rho kinase (ROCK) inhibition with Y-27632 on branching morphogenesis of E12.5 SMGs (n = 12) showed significant increases in the number of clefts per bud (*p* ≤ 0.01) and in buds’ circumference (*p* ≤ 0.05). In addition, numbers of maturing buds per gland were reduced, although these changes were not significant. (**b**) Fluorescence staining of F-actin with rhodamine-phalloidin following treatment of E12.5 SMGs with Y-27632 for 24 h revealed interrupted and condensed F-actin organization ( →) with an accompanying loss of extending ductal paths and formation of structures resembling micro-lumens (➔). (**c**) Quantification of Y-27632 treatment of E13.5 SMGs (n = 8) for 24 h showed significant increases in the numbers of clefts (*p* ≤ 0.0001), reduced numbers of buds per gland (*p* ≤ 0.01) along with increased buds’ circumference (*p* ≤ 0.0001). (**d**) Immunofluorescence staining of ZO-1 in Y-27632-treated E13.5 glands revealed diffuse cytoplasmic distribution of ZO-1 with a loss of its localization to the sites of asymmetry accompanied by interrupted F-actin filaments, detectable micro-lumens (➔) and loss of expansion of cells with asymmetry ( →). (**d’**) Schematic of changes in F-actin organization upon inhibition of ROCK with Y-27632 in E13.5 SMGs highlighting loss of F-actin-demarcated ductal paths and formation of micro-lumens. Size bars, 20 µm.

ROCK signaling has been shown to be involved in regulating epithelial tissue polarity in the developing SMG through activation of the polarity regulator PAR-1b (MARK2), distinct from the phospho-myosin (p-myosin) pathway^39^. When ROCK signaling is inhibited, polarity of the acinar progenitor cells that comprise the outer layer of the bud is lost coincident with aberrant polarization of interior bud cells. Thus, we examined the role of ROCK in setting up and maintaining polarity in the patterning of the ductal tree during SMG development by exposing E12.5 and E13.5 SMGs to the Rho-kinase inhibitor Y-27632 (30 µM) for 24 h in culture and labeling for F-actin and ZO-1. Quantification of morphological changes in response to Y-27632 revealed a dramatic increase in the number of clefts per bud corresponding to increased bud circumference and diminished number of buds (Fig. 7a, bar graphs). Blocking ROCK signaling at E12.5 did not prevent setting up of SCAs, although F-actin was discontinuous (Fig. 7b, Y-27632, arrowheads). Interestingly, in response to Y-27632, cells in the proximal bud regions seemed to be organizing around a shared F-actin-rich domain (Fig. 7b, Y-27632, arrowheads) along with the appearance of microlumens (Fig. 7b, Y-27632, block arrows). Similar to the scenario found with E12.5 SMGs, exposure of SMGs to the ROCK inhibitor at E13.5 resulted in significantly increased number of clefts per bud, diminished number of buds and increased bud circumference (Fig. 7c). These morphological changes were associated with the presence of discontinuous F-actin filaments and aberrant formation of F-actin/ZO-1-rich microlumens throughout the inner bud, suggesting failure to maintain ductal paths across the field in a distal direction (Fig. 7d and schematic Fig. 7d’). Therefore, Rho-kinase is important for the proper temporal sequence of duct formation in the developing SMG but not specification.

**Figure 8:**
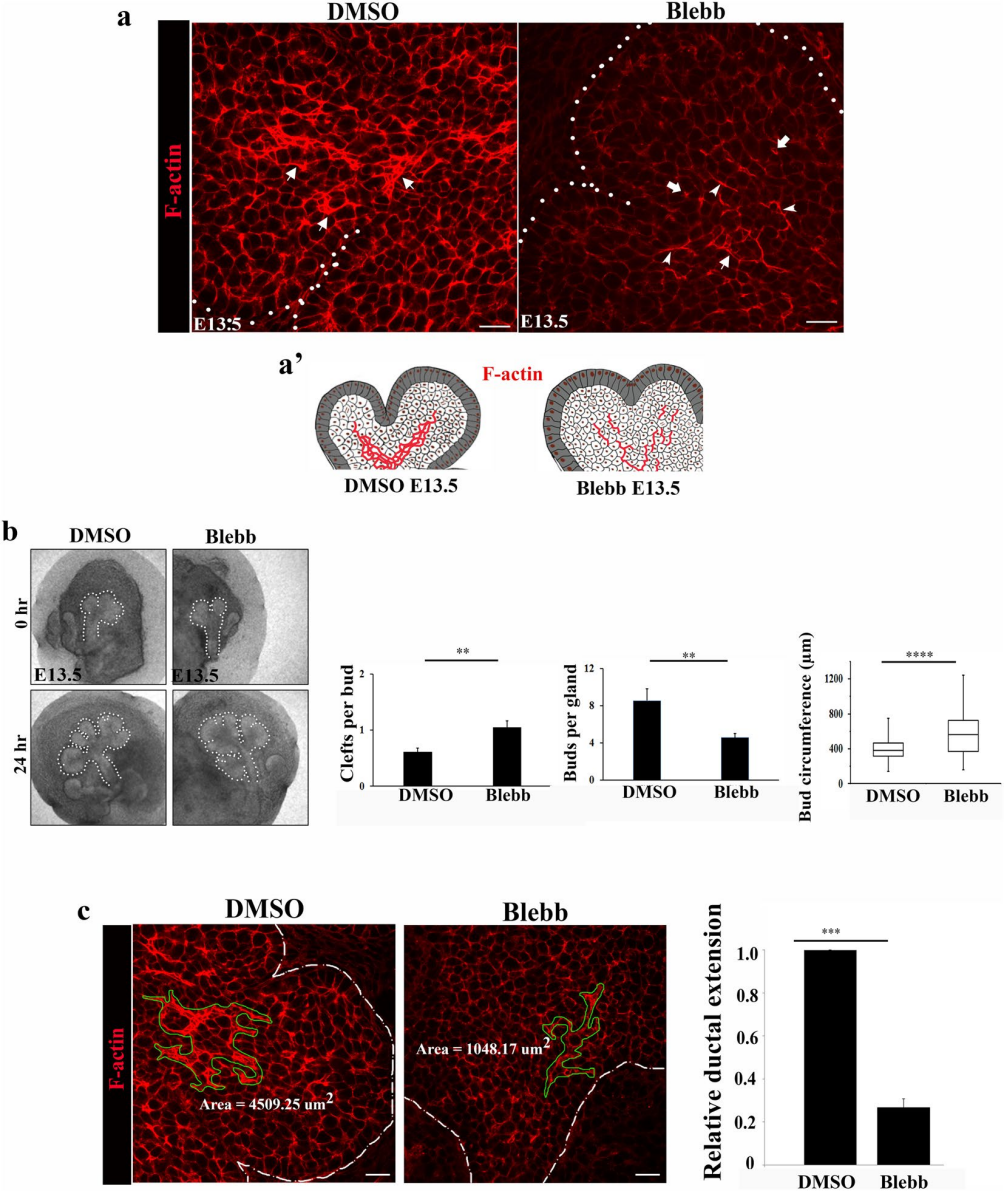
Inhibition of myosin IIB with blebbistatin abolishes F-actin localization to the sites of cellular asymmetry. (**a**) E13.5 SMGs treated with blebbistatin for 24 h displayed greatly reduced and interrupted F-actin staining in the proximal bud regions compared to expanded and continuous distribution of F-actin extending into the newly forming buds in the control DMSO-treated glands ( →). (**a’**) A schematic depicting changes in F-actin distribution from prominent deposition demarcating ductal paths to interrupted filaments. (**b**). Bright field images of the inhibitory effects of blebbistatin on branching morphogenesis of E13.5 SMGs (n = 15). Quantification of morphogenetic changes showed significant increases in numbers of shallow clefts (*p* ≤ 0.01), reduction in bud numbers (*p* ≤ 0.01) and greater bud circumference (*p* ≤ 0.0001). (**c**) Quantification of inhibitory effects of blebbistatin on F-actin distribution (areas defined by green outlines) in proximal bud regions revealed a 3.3-fold reduction in F-actin expansion (bar graph, *p* ≤ 0.001). Size bars, 20 µm.

To examine whether myosin II provides the mechanical forces needed to organize the polarized inner bud cells that have been specified along the ductal paths into mature ductal structures, E13.5 SMGs were treated with blebbistatin, a direct myosin II inhibitor. The SMGs were exposed to the inhibitor in ex vivo culture over 24 h during which time complex cellular rearrangements occur to form classical ductal structures in the control SMGs. Blebbistatin had significant inhibitory impact on F-actin organization. The extensive networks of F-actin typically established by E13.5 in control SMGs failed to reorganize to form ductal structures when myosin II activation was blocked by blebbistatin without impacting apical F-actin asymmetries in presumptive duct cells with prominent aberrations in the branching regions of the developing glands (Fig. 8a and schematic Fig. 8a’). These alterations in F-actin organization were associated with dramatic changes in gland morphologies which revealed significant increases in a number of immature clefts and overall bud circumference, coincident with a reduced number of mature buds (Fig. 8b, bar graphs). Quantification of F-actin spread revealed significant constriction (Fig. 8c). These data indicated that myosin II was not required for the asymmetrical deposition of F-actin but played a central role in the maintenance of ductal extensions.

## Discussion

Branching morphogenesis of the SMG is a complex developmental process requiring precise coordination of ductal cell specification with ductal path extension and with the formation of new buds in the expanding salivary epithelium. Here, we present the first spatiotemporal map of the morphogenetic events of ductal cell specification and duct extension during the early stages of SMG embryonic development. We identify key regulatory molecules and mechanisms by which ductal progenitor cells acquire cellular asymmetries and drive their reorganization into prospective ductal structures in the inner bud. Using ex vivo developing embryonic SMGs and loss-of-function approaches we show that the proximal extension of ducts into the inner bud is specified by assembly of F-actin cytoskeletal structures along a single side of cells in the proximal-to-center regions of the bud, followed by recruitment of ZO-1 to these newly defined sites of cellular asymmetry. Knockdown studies with siRNA demonstrate that ZO-1 plays essential roles in connecting the F-actin-specified cells, as well as in determining the future path of duct formation and the initial convergence of progenitor cells that form the future duct. The discovery of these early F-actin/ZO-1 asymmetries at E12.5 is particularly noteworthy because this is a developmental time that precedes clefting, branching, polarization of ductal cells and the appearance of lumenized ductal structures in the inner bud. Further, many of the cells with F-actin/ZO-1 asymmetries that localize along the field of duct specification are enriched in K5, a cytokeratin expressed by the ductal progenitors that produce WNT, an inductive signal for gangliogenesis that innervates the SMG. Therefore, our results suggest that F-actin and ZO-1 are integral components of the pathway responsible for patterning innervation during SMG development. We note that parasympathetic innervation of the SMG has been shown to regulate the morphogenetic processes of tubulogenesis that precedes lumenization of maturing ducts^7^ and to maintain a reservoir of undifferentiated K5+progenitor cells that are linked to SMG regeneration^5^. Collectively, our studies identify cell polarity cues that guide patterning of a ductal tree during branching morphogenesis of the SMG.

The F-actin/ZO-1 cellular asymmetries that guide the positioning of ductal progenitors within the inner bud at the single bud stage (E12.5) serve as the sites for recruitment of proteins that determine apical-basal polarization of these ductal epithelial cells prior to lumenization of the ducts. At E12.5, a developmental time before polarization of the presumptive ductal epithelium, few inner bud cells are marked by a distinct expression and localization of polarity complex proteins. However, by E13.5, as the cells in the duct-forming region of the inner bud begin to establish apical-basal polarity, there is a notable presence of a polarity protein aPKCζ along F-actin/ZO-1-defined cellular asymmetries that is dependent on ZO-1. This resembles the scenario found in the epidermis of the skin and the regulation of epidermal stratification^40^. By E14.5, when lumenized ductal structures form in the developing SMG, PKCζ is more highly enriched along apical domains along the ductal tree.

During establishment of apical-basal polarity, the polarity proteins are important in defining the apical domains of an epithelial cell, while polarization of the basolateral domain involves signaling through the PAR-1b (MARK2) signaling pathway. Polarization of the outer acinar layer of the developing SMG requires ROCK signaling through PAR-1b^39^. In those studies, the inhibition of ROCK activity prevented organization of the basement membrane along the basolateral zone of the polarized acinar cells, which defines the outer edge of the SMG epithelial bud. Interestingly, ROCK inhibition also caused mislocalization of PAR-1b to cells in the inner regions of the bud leading to aberrant polarization of inner bud cells including assembly of basally localized basement membrane structures within the bud in cells with F-actin-defined apical domains. Our studies confirm the aberrant assembly and collective organization of F-actin-rich domains in the inner bud region in embryonic SMGs exposed to this ROCK inhibitor Y27632 as early as E12.5. These F-actin-rich apical domains of the inner bud cells often formed micro-lumens, indicating that an aberrant morphogenesis was even more extensive when ROCK activity was blocked at E13.5. The observed induction of inner bud microlumen formation in response to ROCK inhibition at this early stage of SMG development parallels results previously reported by Ewald et al. in the developing mammary gland and epithelial MDCK cell models^41,42^. Together, these findings point to an important role for a Rho-kinase signaling pathway in regulating the temporal events during ductal cell polarization to assure that lumen formation does not occur prior to setting the pattern for extending the ductal tree.

While ROCK can be an upstream activator of myosin, our findings and those of others^39^ show that in the developing SMG, ROCK regulates cell polarity through a pathway distinct from its function in the activation of myosin II. In contrast to the effects of the ROCK inhibitor, exposure of E13.5 SMGs to the direct myosin inhibitor blebbistatin prevents further maturation of ducts without affecting F-actin/ZO-1 cellular asymmetries, a result consistent with a role for myosin-driven F-actin contraction in lumen formation^43^. Similar findings were observed when E13.5 SMGs were exposed to ML-7, an inhibitor of myosin light-chain kinase (MLCK), an upstream regulator of myosin phosphorylation (data not shown). Therefore, it is not surprising that at E14.5, myosin IIB is highly localized to cell vertices where the apical domains of multiple ductal epithelial cells link together. While myosin IIB co-localizes with F-actin at the cell vertices, the overall distribution of F-actin is much more extensive, presenting as a contiguous actin-rich domain that connects the apical zones of the epithelial cells that will line the ductal lumen. The localization of myosin IIB to these cells’ apical vertices, which we have also demonstrated is present in its active, phosphorylated form, suggests a role for this F-actin binding motor protein in apical constriction, a process that would be important for morphological rearrangements that bend the apical domains of the epithelial cells during duct maturation^28,35,36,44^. Further, the localization of myosin IIB to cell vertices has been shown to facilitate cellular rearrangements during which cells form new interactive surfaces and acquire additional neighbors^45^.

Collectively, our studies show that the pathway involved in duct cell specification and ductal axis elongation involves asymmetric distribution of F-actin/ZO-1 within a population of K5+ ductal progenitors. Polarity complex protein aPKCζ is recruited to these F-actin/ZO-1 asymmetries to define the apico-basal polarity of interior presumptive duct cells, the temporal occurrence of which is likely regulated by the Rho kinase signaling pathway. With development, this asymmetry continues to expand distally across a field of cells in the duct-forming region. In addition to their role in setting up the pattern of the ductal tree, ZO-1 and aPKCζ also are required for the proper morphogenesis of the SMG. After the duct-forming region is specified and prospective ductal epithelial cells become polarized, apical constriction forces generated by active myosin contribute to duct maturation. The mechanisms revealed in these studies provide clues to events likely to play important roles in the regeneration of SMGs and to serve as a basis for the elucidation of regulators of duct formation in other epithelial tissues that develop through branching morphogenesis.

## Methods

### SMG cultures

Studies described in this report utilized pregnant CD-1 mice purchased from the Charles River Laboratories. Submandibular and sublingual gland (SMG) rudiments were dissected from mouse embryos at embryonic (E) stages E12.5, E13.5, E14.5, and grown ex vivo on Whatman Nuclepore Track-etch filters under designated conditions according to standard procedures, as described by us and others^1–3,20^. All mouse studies were performed according to the Boston University Medical Campus (BUMC) Institutional Animal Care and Use Committee (IACUC) Ethical Guidelines^3,20^.

### Reagents

For immunofluorescence analyses, antibodies specific for ZO-1, keratin 5, PKCζ, myosin II and phospho-myosin were purchased from BD Transduction Laboratories. Secondary antibodies included AffiniPure goat anti-mouse and goat anti-rabbit IgG Fab fragments from either Jackson ImmunoResearch Laboratories or from Molecular Probes. Localization and distribution of F-actin was followed by the staining of SMGs with Rhodamine- or Alexa-conjugated phalloidin^3,20^, while nuclei were visualized with monomeric cyanine nucleic acid stain, both from Molecular Probes. Evaluation of transfection efficiency was carried out with Cy3-siRNA, obtained from Dharmacon.

### RNA interference

For functional perturbation of ZO-1 expression, siRNA was obtained from Dharmacon, while a non-targeting control was from Qiagen. Based on the initial determination of optimal inhibitory siRNA dose and time of treatment, concentration of 400 nM siRNA for 22–48 h was selected. The experimental design involved transfection of one gland from a pair of E12.5 or E13.5 SMGs with ZO-1 siRNA (S) while the second gland was designated for transfection with a non-silencing (NS) control using RNAiFect (Qiagen). To assure statistical significance, six SMG rudiments were cultured per filter for a total of three filters per condition, with each experiment being repeated independently at least three times. Transfection efficiency was assessed by tracking siRNA uptake by staining SMGs with Cy3-siRNA (Dharmacon). The effect of ZO-1 siRNA on the ZO-1 steady state transcript levels was measured using total RNA isolated from pooled SMGs from three independent experiments, according to standard procedures^20^. ZO-1 transcript levels were determined by RT-PCR using 29S as a normalizing control^20^.

### Immunofluorescence

Cultured SMG rudiments from different experimental conditions were processed for immunofluorescence analysis as described by us previously^20^. Briefly, at the completion of each experimental design/treatment, SMGs were fixed in 3.7% paraformaldehyde for 2 h. The glands were then washed four times in PBS followed by permeabilization with 0.1% Triton X-100 for 15 min at room temperature. Alternatively, in order to better visualize cytoskeletal association of ZO-1 and PKCζ, treatment with ice-cold acetone/methanol (1:1) for 15 min replaced the 3.7% paraformaldehyde fixation, followed by four washes with PBS. Next, the glands were blocked with 10% donkey serum and 1% BSA in 0.1% PBS-Tween 20 overnight, followed by incubation with selected primary antibodies for 4 h at room temperature. Signal visualization was carried out by staining the SMGs for 1 h with fluorescein-derivatized either donkey or goat F(ab)2 fragments secondary antibodies. In order to assess F-actin organization, phalloidin conjugated to either Rhodamine or Alexa Fluor was added for 30 min, while monomeric cyanine nucleic acid stain was used to visualize the nuclei.

### Confocal image analysis

High-resolution imaging of stained SMGs was carried out with a Zeiss confocal laser scanning microscope LSM510 META and images were processed using LSM510-expert mode acquisition software^20^. Z-stacks with the 40X objective were acquired with a single optical slice of 0.5 microns. Images are shown as a single optical slice from a z-stack, orthogonal projection, or x–y orthogonal view through the entire gland. 3D images were created with Imaris 3D view.

### Image quantification, morphometric and statistical analyses

Analyses of imaging data were performed using Imaris for Cell Biologists by Bitplane with Imaris Cell for analyses of 2D and 3D images. MeasurementPro was used for quantification and statistics software. Data are presented as mean + s.e.m. Student’s t-test for comparison of two groups and analyses of variance was used to assess statistical significance, while Bonferroni’s post-test was used for analyses carried out with Prism 5 software.

Quantification of the area of duct extension based on F-actin labeling was examined in response to treatments with either ZO-1 siRNA or pharmacological inhibitors. This quantification was carried out by image capture, measurement and processing using the NIS Elements imaging software (Nikon Instruments). Changes in bud geometries in response to treatments with inhibitors were quantified using 5X immunofluorescence images of multiple salivary glands (n = 15–20) from 4 treatment groups: DMSO, ML-7, Bleb, and Y-27632. Buds and clefts of each immunofluorescence image were hand-outlined on inverted grayscale images. Their dimensions were measured in pixels using Adobe Illustrator image editing software and were converted into micrometers using a formula calculated from corresponding scale bars (distance measured in pixels × 1.75 µm/pixel). The values were then used to generate box plots with the Excel software, and statistical analyses were performed using Student’s t-test.
